# Photosynthetic responses of large old *Zelkova serrata* (Thunb.) Makino trees to different growth environments

**DOI:** 10.1038/s41598-023-47561-3

**Published:** 2023-11-18

**Authors:** Ji Sun Jung, Gwang Gyu Lee, Ji Won Son, Chae Won Kim, Yoo Jin Ahn

**Affiliations:** 1 Natural Heritage Center, National Research Institute of Cultural Heritage, Daejeon, Republic of Korea; 2 Korea National University of Cultural Heritage, Buyeo, Republic of Korea

**Keywords:** Urban ecology, Plant ecology, Plant physiology

## Abstract

Large old trees, which provide ecosystem services and serve as a historical and cultural heritage, are exposed to various environmental threats, such as habitat fragmentation and climate change, necessitating diagnosis of tangible and intangible stresses and their effects on tree growth for effective management. This study investigated the photosynthetic characteristics of 25 large old *Zelkova serrata* (Thunb.) Makino trees in Chungcheong Province, Korea, and identified the physical environmental factors affecting their physiological responses. Maximum assimilation rate (A_max_) was the highest in July (summer), transpiration rate (E) and stomatal conductance (g_s_) increased from May (spring) to September (fall), and water use efficiency (WUE) was the highest in May (spring) and decreased until September (fall). A_max_ decreased as tree height increased. Ambient CO_2_ and vapor pressure deficit (VPD) were negatively correlated with photosynthetic parameters throughout the growth season and in July (summer) and September (fall), respectively. Physical environmental factors exhibited complex effect on physiological activities, which increased with wide growth space and decreased with deep soil covering and high impervious ground surface ratio. Physiological responses differed with surface types within the growth space, with bare land showing higher mean A_max_, E, and g_s_ than areas with mulching material or concrete. This study quantitatively determined the physiological activities of large old *Z. serrata* and proposes appropriate management measures for ensuring their healthy growth in abiotic stress environment.

## Introduction

Large old trees are valuable natural treasures that provide valuable ecosystem and landscape services, as well as cultural and historical significance unique to each nation. They serve as biotopes that significantly impact water and nutrient cycles, as well as the habitats of various fungi, plants, and animals^1^. Urbanization and climate change, pose threats to large old trees, including water scarcity, pests and pathogens, and habitat fragmentation. Unlike general trees, large old trees have developed under past climatic conditions conducive to their growth, making them potentially vulnerable or poorly adaptable to rapid environmental changes.

In urban areas, large old trees are particularly susceptible to abiotic damage such as dryness, high temperatures, foot pressure, and soil covering. These factors cause growth stress and negatively affect crucial physiological mechanisms in trees, including leaf gas exchange ability^2, 3^. Additionally, the size of large trees limits their ability to transport sap against gravity and cell resistance, leading to hydraulic failure when compensating for water loss through transpiration^1, 4^.

Conducting research to analyze photosynthetic responses is essential for objectively assessing the health of trees before permanent damage occurs due to the climate crisis and unfavorable urban growth environment. Photosynthetic responses are fundamental and intricate physiological processes in green plants. Many environmental factors, such as temperature, light, atmospheric CO_2_, humidity, and soil moisture, can profoundly affect photosynthesis, and damage can impair overall photosynthesis capacity^5^. Characteristics like net assimilation rate, stomatal conductance, and transpiration rate in response to light and CO_2_ have proven crucial for understanding how plants adapt their photosynthetic process to changing environments^6–8^.

Despite extensive knowledge about trees and their photosynthetic responses, most studies have focused on general trees, leaving a gap in our understanding of large old trees. Age-related changes in these trees encompass reduced rates of carbon assimilation and growth across all organs, along with increased susceptibility to disease, insects and other stresses^9^. Older, mature trees also experience decreased net productivity, due to declining meristematic activity, resulting in reduced leaf renewal and transportation difficulties caused by the structural complexity associated with numerous meristematic organs^10^. Gower et al.^11^ attributed the decrease in net primary production to an altered balance between photosynthesis and respiration with stand age, reduced soil nutrient availability, and increased stomatal limitation. Thus, it is expected that photosynthesis differs between old trees and relatively young trees.

*Zelkova serrata* (Thunb.) Makino is a deciduous broadleaf tree in the elm family widely distributed throughout East Asia, including Korea, China, and Japan. In China, they are native to river basins and dense forests at 500 to 1900 m above sea level in the South of the Qinling Mountains and Huai River basins^12^. In Korea, a substantially high percentage of large old *Z. serrata* trees has been designated as natural monuments. In China, *Z. serrata* trees have been designated as national second-class key protected plants owing to decreasing numbers of native trees^13^.

Studies have examined the photosynthetic characteristics of *Z. serrata*, focusing on seedlings and their physiological responses to light, moisture, and growth density^14–16^. However, there is a lack of clear understanding regarding the general photosynthetic characteristics of large old *Z. serrata* trees, which typically exceed 200 years of age and grow in natural settings. Furthermore, negligible research has been conducted to identify the specific factors within diverse growth environments that influence the photosynthetic responses of these large old *Z. serrata* trees. It is crucial to address these gaps in knowledge, especially considering the potential impact of chronic stress caused by habitat fragmentation and urbanizations on the long-term survival of high-value, large old *Z. serrata* trees. Therefore, investigating the physiological responses of large old *Z. serrata* trees in different environmental conditions is urgently required.

The objectives of this study were to (1) investigate basic data on the photosynthetic physiological activities of large old trees, focusing on large old *Z. serrata* under central cool temperate climate and (2) identify the growth environmental factors that affect photosynthetic physiological responses of large old *Z. serrata*. The findings of the study can enhance the knowledge about the photosynthetic physiological responses of large old *Z. serrata* trees and provide important insights for policies on improving the growth environment to protect heritage trees.

## Results

### Photosynthetic responses of large old *Z*. *serrata*

**Figure 1:**
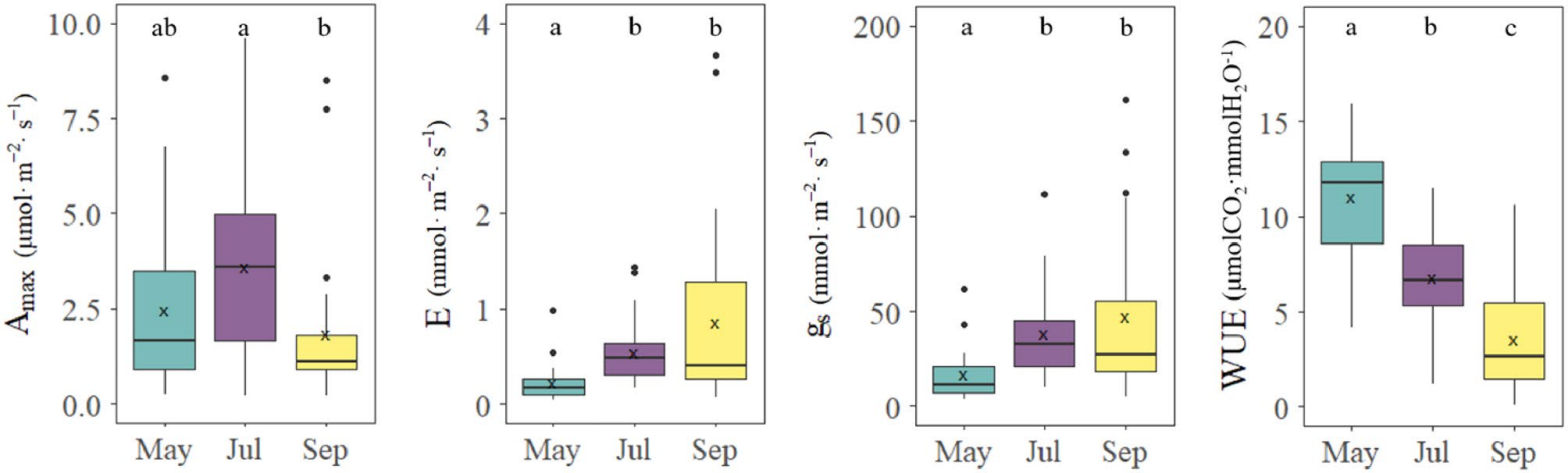
Differences in photosynthetic parameters of large old *Z. serrata* by month. Post-hoc test results are indicated by lowercase letters and different letters indicate significant differences between groups at the significance level of p < 0.001.

Photosynthetic responses of large old *Z. serrata*, such as maximum assimilation rate (A_max_), transpiration rate (E), stomatal conductance (g_s_) and water use efficiency (WUE), showed different patterns as the seasons changed. The mean A_max_ during the entire growth season (May–September) was 2.73 ± 1.65 μmol·m^–2^·s^–1^, with the highest value of 3.93 ± 2.92 μmol·m^–2^·s^–1^ found in July (summer), followed by 2.47 ± 2.09 μmol·m^–2^·s^–1^ in May (spring) and 1.86 ± 2.05 μmol·m^–2^·s^–1^ in September (fall) (p = 0.0037). The mean E during the growth season was 0.56 ± 0.40 mmol·m^–2^·s^–1^, which gradually increased after May (spring) (0.23 ± 0.20 mmol·m^–2^·s^–1^) and reached the highest value in September (fall) (0.86 ± 0.97 mmol·m^–2^·s^–1^) (p < 0.001). The mean g_s_ during the growth season was 41.90 ± 32.01 mmol·m^–2^·s^–1^, and similar to the E value, the lowest value was observed in May (spring) (16.69 ± 13.61 mmol·m^–2^·s^–1^), which tended to gradually increase to reach the highest value in September (fall) (67.60 ± 81.79 mmol·m^–2^·s^–1^) (p < 0.001). Mean water use efficiency (WUE) was 7.03 ± 1.55 μmolCO_2_·mmolH_2_O^–1^, with the highest value observed in May (spring), during the dry season (11.03 ± 3.11 μmolCO_2_·mmolH_2_O^–1^), followed by a pattern of continued decrease up to September (fall) (3.58 ± 2.74 μmolCO_2_·mmolH_2_O^–1^) (p < 0.001) (Fig. 1).

**Figure 2:**
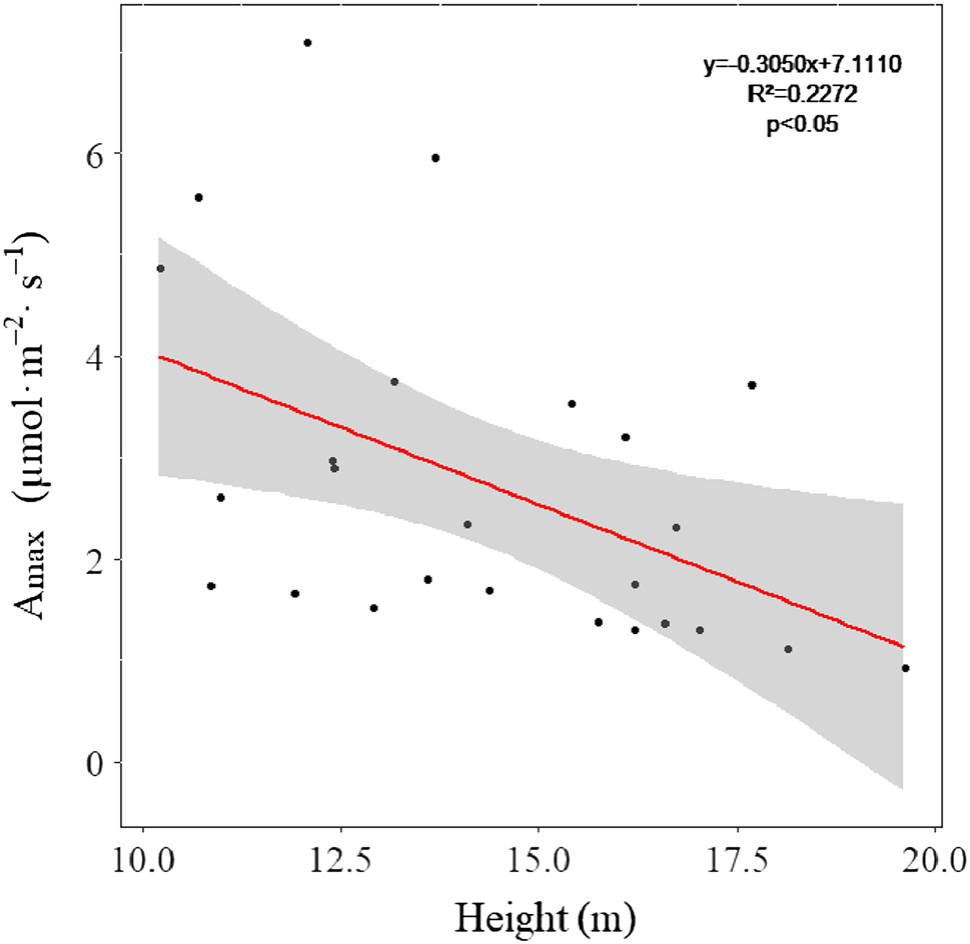
Linear regression graph of maximum assimilation rate (A_max_) according to the tree height of large old *Z. serrata* during the entire growth season (N = 25). Shaded regions represent a 95% confidence interval.

Moreover, there were differences in the photosynthetic responses according to tree height. In the linear regression analysis on physiological activity parameters according to tree height, DBH, and crown width of large old trees during entire growth season (May–September), the results showed that A_max_ decreased with increasing tree height (R^2^ = 0.23, F = 6.76, p < 0.05) (Fig. 2  and Supplementary Table S1). However, DBH and crown width showed no significant differences according to the photosynthetic response characteristics.

### Climatic factors affecting photosynthetic parameters

Investigation of the correlations among photosynthetic parameters according to climatic environmental factors for each month and the entire growth season of large old *Z. serrata* were performed. Moreover, A_max_, E, and g_s_ decreased as photosynthetic active radiation (PAR) increased (r = − 0.53, p < 0.01; r = − 0.44, p < 0.05; r = − 0.43, p < 0.05) and as ambient CO_2_ concentration increased (r = − 0.86, p < 0.01; r = − 0.55, p < 0.01; r = − 0.56, p < 0.01) throughout the entire growth season. Vapor pressure deficit (VPD) was negatively correlated with A_max_ and g_s_ during July (summer) (r = − 0.56, p < 0.01; r = − 0.40, p < 0.05) and A_max_, E, and g_s_ during September (fall) (r = − 0.42, p < 0.05; r = − 0.55, p < 0.01; r = − 0.55, p < 0.01), confirming that it was a factor that had a major impact on stomatal opening and closing under hot and dry conditions. Moreover, VPD showed a high negative correlation with WUE (r = − 0.63, p < 0.01) and a high positive correlation with intercellular CO_2_ (C_i_) during July (summer) (r = 0.56, p < 0.01). WUE showed a high negative correlation with C_i_ during all seasons (r = − 0.97, p < 0.01; r = − 0.99, p < 0.01; r = − 1.00, p < 0.01) and throughout the growth season (r = − 0.99, p < 0.01) (Tables 1 and 2).

**Table 1:**
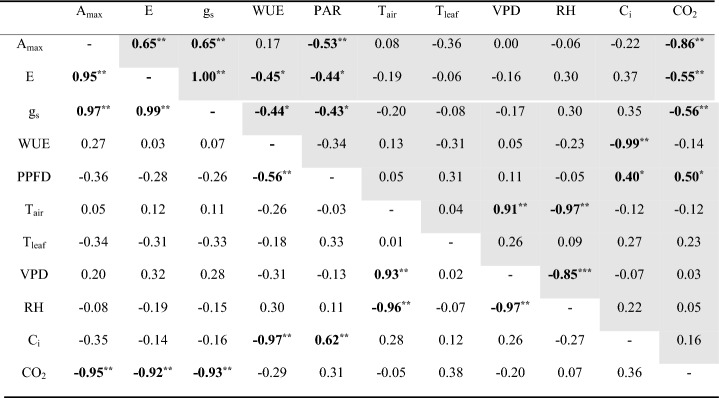
Correlation coefficients between photosynthetic parameters of large old *Z. serrata* and climatic environmental factors during the entire growth season and May (spring).

*PAR* photosynthetically active radiation, *T*_air_ air temperature, *T*_leaf_ leaf temperature, *VPD* vapor pressure deficit, *RH* relative humidity, *C*_i_ intercellular CO_2_, *CO*_2_ ambient CO_2_ during the entire growth season (gray) and May (spring) (white).

*p < 0.05, **p < 0.01.

Significant values are in bold.

**Table 2:**
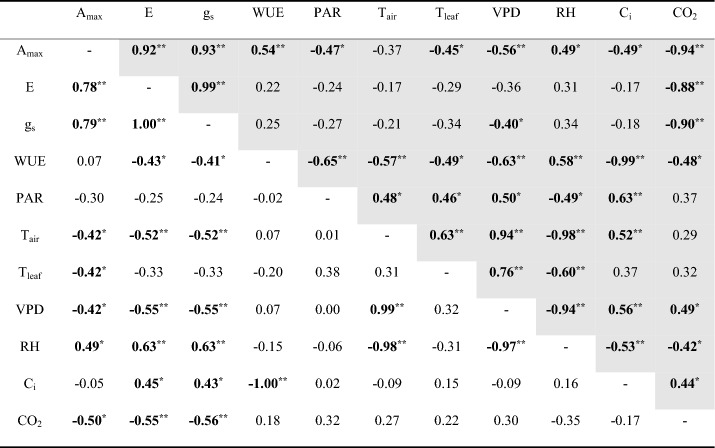
Correlation coefficients between photosynthetic parameters of large old *Z. serrata* and climatic environmental factors during July (summer) and September (fall).

*PAR* photosynthetically active radiation, *T*_air_ air temperature, *T*_leaf_ leaf temperature, *VPD* vapor pressure deficit, *RH* relative humidity, *C*_i_ intercellular CO_2_, *CO*_*2*_ ambient CO_2_ during July (summer) (gray) and September (fall) (white).

*p < 0.05, **p < 0.01.

Significant values are in bold.

### Physical environmental factors affecting photosynthetic parameters

Difference between photosynthetic responses and physical environmental factors (depth of soil covering, impervious surface ratio, growth space ratio, and separation distance from the road) of large old *Z. serrata* were investigated. Correlation coefficients between physical environmental factors and photosynthetic parameters showed that growth space ratio was positively correlated with A_max_ and g_s_ in May (spring), and A_max_ and E in July (summer), whereas impervious surface ratio and depth of soil covering were negatively correlated with A_max_, E, and g_s_, especially during July (summer) (Table 3).

**Table 3:** Correlation coefficients between physical environmental factors and photosynthetic parameters of large old *Z. serrata* in each season.

Season	Parameters	Mean separation distance from the road	Depth of soil covering	Impervious surface ratio	Growth space ratio
May (spring)	A_max_	0.42	− 0.28	− 0.23	0.49*
	E	0.28	− 0.22	− 0.15	0.40
	g_s_	0.35	− 0.26	− 0.20	0.47*
	WUE	0.31	− 0.04	0.02	− 0.01
July (summer)	A_max_	0.18	− 0.45*	− 0.51*	0.46*
	E	0.26	− 0.52**	− 0.50*	0.41*
	g_s_	0.24	− 0.52**	− 0.51*	0.39
	WUE	− 0.03	− 0.10	− 0.22	0.34
September (fall)	A_max_	− 0.15	0.09	0.17	0.08
	E	− 0.08	− 0.03	− 0.03	0.06
	g_s_	− 0.09	− 0.03	− 0.01	0.05
	WUE	− 0.18	0.20	0.34	− 0.07

*p < 0.05, **p < 0.01.

Significant values are in bold.

In the simple linear regression analysis between photosynthetic parameters and physical environmental factors of large old *Z. serrata*, the results showed that A_max_ was significantly linearly related to growth space ratio, depth of soil covering, and impervious surface ratio during July (summer). It tended to increase as the growth space became wider (R^2^ = 0.21, F = 6.06, p < 0.05) and decrease as the depth of soil covering (R^2^ = 0.20, F = 5.92, p < 0.05) and impervious surface ratio (R^2^ = 0.26, F = 7.05, p < 0.05) increased. Similar to A_max_, E increased as the growth space ratio increased (R^2^ = 0.16, F = 4.53, p < 0.05) and decreased as the depth of soil covering (R^2^ = 0.28, F = 8.73, p < 0.01) and impervious surface ratio (R^2^ = 0.25, F = 6.83, p < 0.05) increased. Likewise, the g_s_ decreased as the depth of soil covering (R^2^ = 0.27, F = 8.31, p < 0.01) and impervious surface ratio (R^2^ = 0.26, F = 7.18, p < 0.05) increased during July (summer). There was significant differences between g_s_ and the growth space ratio only during May (spring) (R^2^ = 0.22, F = 6.21, p < 0.05).

**Figure 3:**
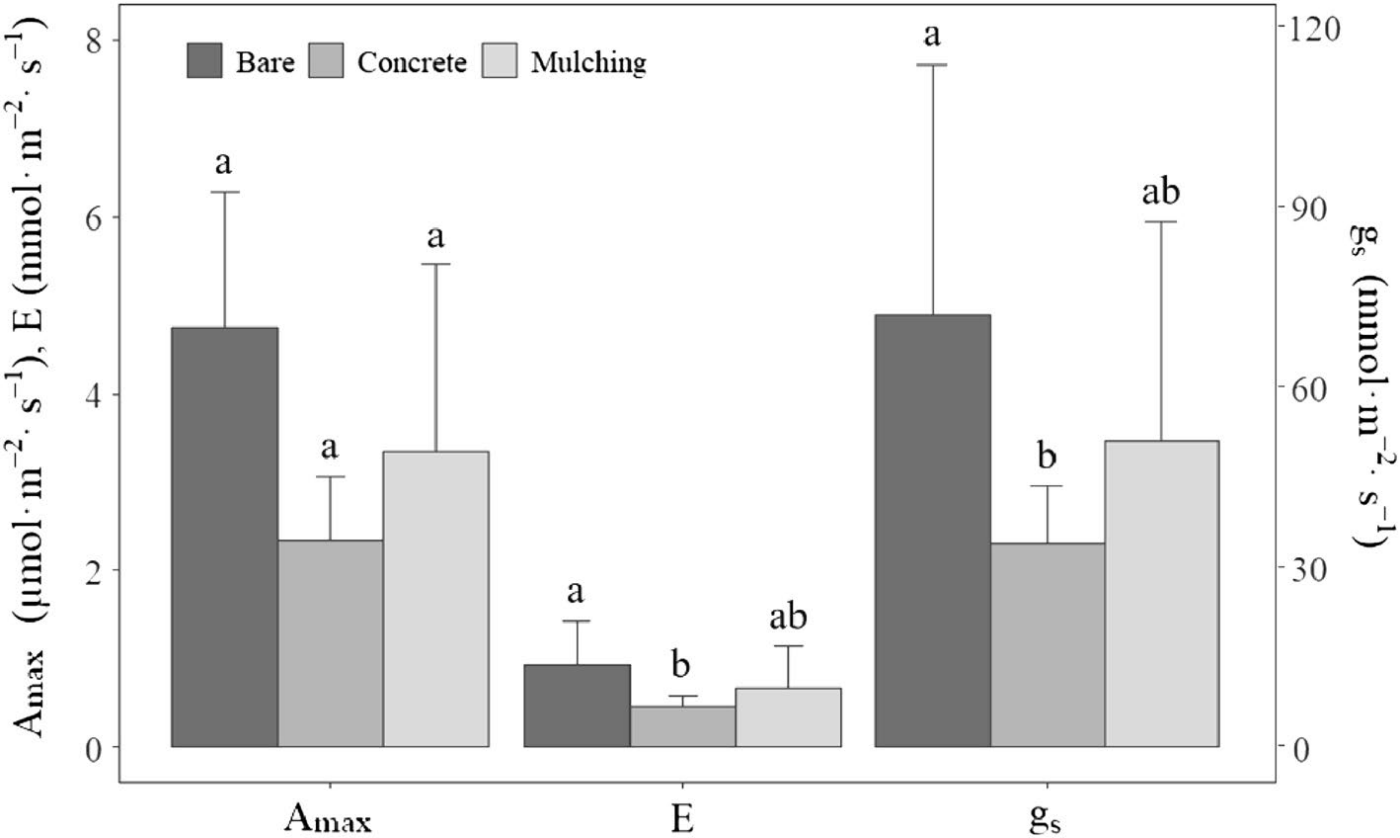
Differences in photosynthetic parameters of large old *Z. serrata* according to surface coverage type during the entire growth season. Post-hoc test results are given in lowercase letters. Different letters indicate a significant difference between groups within a 95% confidence level.

Meanwhile, the Wilcoxon rank-sum test results showed a significant difference in mean A_max_ between the two groups,with 3.00 ± 1.67 μmol·m^–2^·s^–1^ for sites wider than crown width and 1.33 ± 0.17 μmol·m^–2^·s^–1^ for sites narrower than crown width (p < 0.05). Specifically, Kruskal–Wallis H test results showed that there were differences in the photosynthetic responses between groups according to the surface coverage type, categorized as bare land, mulching (e.g. woodchip and gravel), and concrete, within the growth space during the entire growth season. Bare study sites showed the highest mean A_max_ with 4.75 ± 1.52 μmol·m^–2^·s^–1^, followed in order by mulching material (3.33 ± 2.13 μmol·m^–2^·s^–1^) and concrete (2.33 ± 0.73 μmol·m^–2^·s^–1^). The mean E and g_s_ in bare study sites were 0.94 ± 0.50 mmol·m^–2^·s^–1^ and 72.02 ± 41.87 mmol·m^–2^·s^–1^, respectively, which were higher than those of mulching (0.68 ± 0.47 mmol·m^–2^·s^–1^ and 51.16 ± 36.50 mmol·m^–2^·s^–1^, respectively) and concrete (0.47 ± 0.13 mmol·m^–2^·s^–1^ and 34.06 ± 9.46 mmol·m^–2^·s^–1^, respectively) throughout the entire growth season (Fig. 3).

The post-hoc test results in E and g_s_ showed that differences between surface coverage types all appeared to be due to differences between bare land and concrete (p < 0.05).

## Discussion

The maximum assimilation rate (A_max_) of large old *Z. serrata* during July (summer) (3.93 ± 1.22 μmol·m^–2^·s^–1^) was approximately half of the 7.1 μmol·m^–2^·s^–1^ value reported for mature *Z. serrata* (mean DBH: 20 cm; mean tree height: 8 m)^17^ and approximately one-third of the 9 μmol·m^–2^·s^–1^ reported for young *Z. serrata* in similar central cool temperate zones^18^. These findings confirm that the assimilation rate of large old trees tended to be lower than that of young trees.

Seasonally, A_max_ increased from May (spring) to July (summer) to reach the peak value during July (summer) and subsequently showed the lowest value in September (fall) when the air temperature decreased. The maximum mean monthly temperature was recorded in July (summer), followed by May (spring) and September (fall), showing a similar trend as the A_max_. Temperature and photosynthetic rate of *Z. serrata* are known to be significantly correlated^15^ and, in this study, a negative correlation between photosynthetic rate and air temperature in September (fall) was confirmed (p < 0.05). The water use efficiency (WUE) was the highest in May (spring), which is in the dry season. The cumulative precipitation in May (spring) was 8.1 mm, which was the lowest among all months, while the mean air temperature in March and April was 2.3 ℃ higher than the national average for average years. These findings confirm the physiological responses used by trees to lower transpiration during the dry season to increase WUE and reduce the amount of water required for carbon fixation^19, 20^.

In terms of physical traits, differences existed in the photosynthetic responses of large old *Z. serrata*. Generally, as trees mature and their height increases, the distance between the absorption organ (roots) and transpiration organ (leaves) increases, which causes a decrease in the water potential owing to a delay in water flow^21^. In this study, the height of large old *Z. serrata* ranged from 10.2 to 19.6 m, and, A_max_ decreased as tree height increased-. Large old *Z. serrata* in this study is relatively high compared to mature *Z. serrata* trees habituated in the city in a similar climatic zone, while A_max_ of these large old *Z. serrata* is only half of that of mature *Z. serrata*^17^. These findings can be attributed to the characteristics of *Z. serrata* having high height and wide crown width, causing a decrease in the efficiency of sap flow owing to multiple cavitations and a decrease in intravascular pressure with the xylem being extended, which negatively affect the overall photosynthetic mechanism^22, 23^.

Vapor pressure deficit (VPD) is known to have a significant impact on transpiration rate (g_s_) and photosynthetic rate by affecting leaf gas exchange function and CO_2_ assimilation^24, 25^. In this study, VPD had a negative effect on the photosynthetic parameters (A_max_, E, and g_s_) of large old *Z. serrata* during July (summer) and September (fall) and showed a significant correlation with air temperature, which indicates that the environmental conditions surrounding large old *Z. serrata* can lead to unfavorable situations in the phase of climate environment change with increasing high temperature and drought in near future. Moreover, the correlation between VPD and WUE during July (summer) in this study was consistent with the results of the study by Gillner et al.^26^, which showed a decreasing trend in WUE with increasing VPD in tree species under extreme water stress at a temperature of 25 ℃. This is believed to be attributed to increased water loss from the plants due to high VPD, which increased water stress and potentially affected the reduction of WUE^27^.

**Figure 4:**
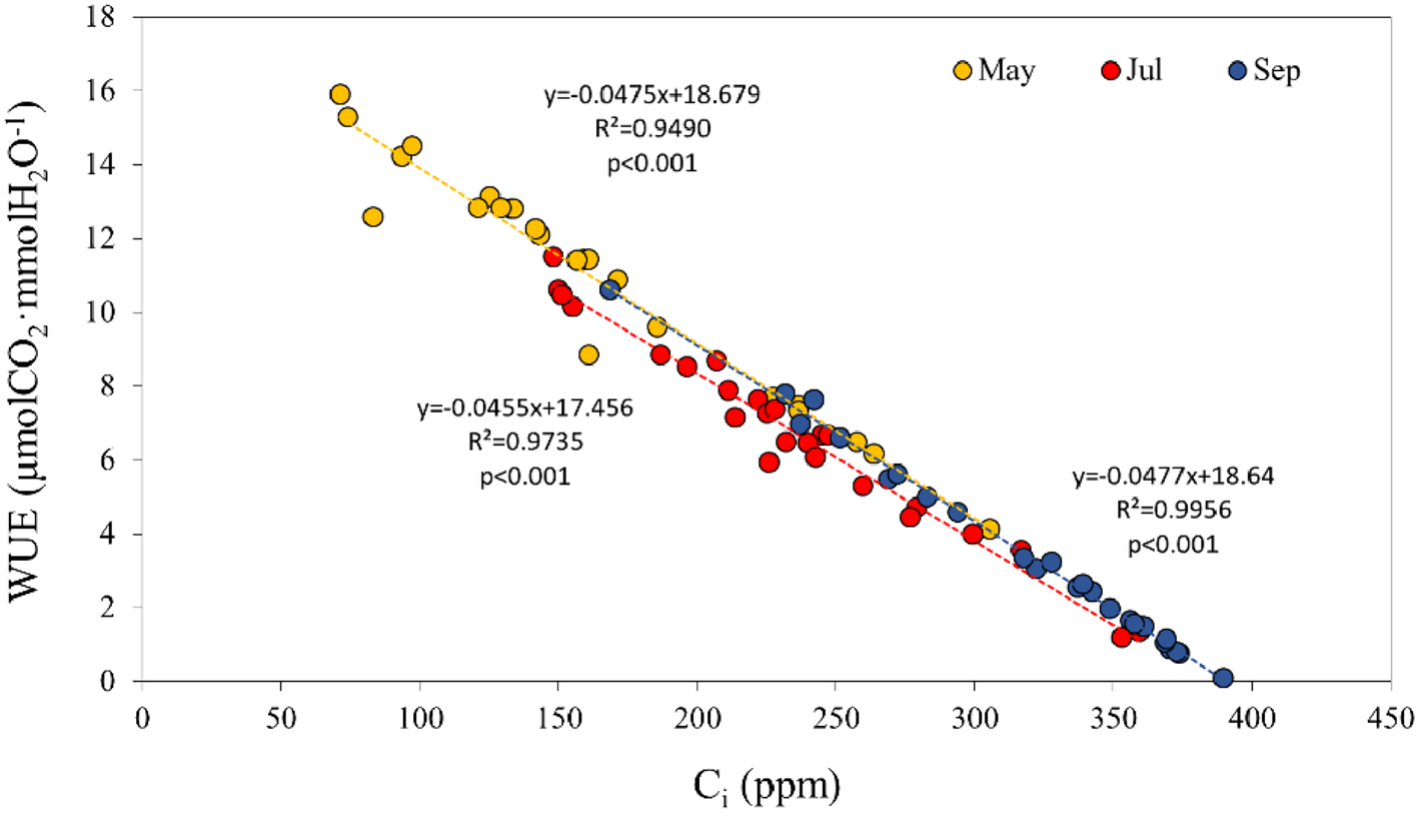
Seasonal changes in water use efficiency (WUE) of large old *Z. serrata* according to intercellular CO_2_ concentration (C_i_).

When plants are exposed to water stress or respiratory rate increases under high-temperature conditions, CO_2_ concentration in leaves increases, leading to stomatal closure, and such an increase in CO_2_ concentration increases the WUE^19^. However, WUE is significantly influenced by environmental conditions and shows a different pattern depending on the tree species^28, 29^. Moreover, extreme water stress severely limits net photosynthesis to cause a decrease in WUE^26, 30^, which may be attributable to severely restricted CO_2_ and water supply due to stomatal closure, as well as increased intracellular CO_2_ diffusion resistance due to mesophyll resistance^29, 31^. Trees in highly covered locations, such as urban centers, generally have a low leaf gas exchange rate depending on the drought level but only a slight change in the WUE^30^. These findings suggest that large old *Z. serrata* in similar environments showed a decrease in WUE owing to a decrease in the efficiency of CO_2_ used in assimilation caused by diffusion resistance despite high g_s_ and an increase in C_i_ from May (spring) to September (fall) (Fig. 4).

In addition, among different physical environment factors, photosynthetic response of large old Z. serrata showed significant correlations with the impervious surface ratio, depth of soil covering, growth space ratio and surface coverage type. Gillner et al.^2^ performed a tree-ring analysis and found that trees growing in enclosed environments covered by non-porous material showed differences in growth according to field conditions compared to trees growing in less-enclosed environments. Various studies have found that impervious pavement inhibits the photosynthesis of trees and negatively affects the biomass and growth of pine, ash, and maple trees^32–34^. In contrast, the impervious pavement causes a significant decrease in A_max_ and the photosynthetic rate of maple trees^3^. Large old *Z. serrata* also showed decreases in A_max,_ E, and g_s_, especially during July (summer), as the impervious surface ratio of growth space increased, with A_max_ decreasing throughout the entire growth season, except in September (fall). Generally, great increases in air temperature and low soil moisture are found in areas with impervious pavements^3, 32^. A sustained water deficit can lead to increased photoinhibition, especially during periods with high temperatures and strong sunlight, such as July (summer), which causes a further decrease in net photosynthesis^35^. The impervious soil environment in the study sites appeared to cause desiccation stress in large old *Z. serrata* trees, which in turn caused an overall decline in photosynthetic efficiency.

The findings of the present study also showed decreases in A_max,_ E, and g_s_ in July (summer), the month of highest photosynthesis for large old *Z. serrata*, as the depth of the soil covering increased (Table 3). Soil covering can inhibit root growth by interfering with oxygen supply to the root collar area, which has high root density and respiration, ultimately leading to a decline in tree growth. Over-mulching or buried root flare due to planting the tree too deep can reduce gas exchange in the soil to kill inner bark tissues, which leads to reduced absorption of water and nutrients that influence the survival of the plant. In particular, oxygen deprivation due to over-mulching is known to increase when active root growth occurs or during humid periods^36^.

In this study, there was also a significant difference in that carbon assimilation decreased according to the depth of soil covering during July (summer) with high humidity. In the present study, A_max_, E, and g_s_ increased as the growth space ratio of large old *Z. serrata* increased, which influenced all photosynthetic parameters, especially during July (summer). Moreover, study sites with a narrower growth space than the crown width showed lower A_max_ during the entire growth season than study sites wider than the crown width. In a similar study on the effects of limited planting space on tree activities and growth, roadside trees planted in areas with width < 1.25 m showed low resilience against stress and growth level, supporting various studies that reported limited growth space being associated with poor tree growth^37–39^. This phenomenon may be owing to limited growth space not providing enough space for roots to spread out and interfering with air, water, and nutrient flows, accompanied by increases in the severity and likelihood of damage as the tree diameter increases^40^. The present study also showed differences in photosynthetic responses according to the specific surface type of growth space. Surface coverage can have a negative effect on photosynthetic parameters, including A_max_, E, and g_s_, by causing desiccation stress in trees^3, 41^. Furthermore, concrete-covered study sites showed lower A_max_, E, and g_s_ during the entire growth season than bare study sites and mulching material-covered study sites (Fig. 3).

Various physical environmental factors had a combined effect on the photosynthetic physiological activities of large old *Z. serrata*, especially during July (summer) when high temperatures are maintained. Deep soil covering, a narrow growth space, and a high impervious surface ratio influence the physiological activities of large old *Z. serrata* by creating microclimates and soil environments unfavorable for photosynthetic activities. Large old trees growing close to urban centers or towns are at high risk of being exposed to abiotic damage, such as surface pavement. In particular, large old *Z. serrata* have lower physiological activities than young *Z. serrata* and lower water availability owing to their height, which likely reduces their ability to cope with unfavorable growth environments. Moreover, hot and dry growth conditions, such as in the study sites, can worsen owing to climate change, thereby increasing the likelihood of high VPD in the future, unfavorably influencing the photosynthesis of large old *Z. serrata*. Therefore, fundamental improvement in soil aeration, including removing soil coverage within the growth space and expanding the growth space to be at least as wide as the crown width, is proposed for the healthy management of large old *Z. serrata*. In particular, leaving the surface of growth space bare is ideal; however, in areas with concerns of soil compaction, appropriate mulching materials, rather than concrete, need to be considered to increase soil nutrient retention and to support the biological activities of soil that affect the rhizosphere^42^.

The findings of this study can provide insights for the management and conservation of large old *Z. serrata* trees in similar environments. The recommendations, such as improving soil aeration, expanding growth space, and using appropriate mulching materials, can guide practices to enhance the health and vitality of these trees. Also, the study highlights the potential effects of climate change, particularly increasing temperatures and drought conditions, on the photosynthetic activity of large old trees. The findings can contribute to the understanding of how climate change may impact the physiological responses of trees in similar cool temperate zones. Furthermore, the study emphasizes the negative effects of impervious surfaces, soil covering, and limited growth space on the photosynthetic vitality of large old trees. The findings can inform urban planners and designers about the importance of preserving adequate space and minimizing soil and surface coverings to maintain healthy urban tree populations.

Focusing on various physical environmental factors, this study examined the effect of individual factors on the photosynthetic vitality of large old *Z. serrata*, which is the result of a combination of various variables, such as physical environmental variables, meteorological environmental factors, and individual size specifications. Also, while the study examined various physical environmental factors, it may not have accounted for all potential variables that could influence the photosynthetic vitality of large old *Z. serrata*. Factors such as pollution levels, soil composition, and microclimatic variations, and their influence on the results need to be assessed. Besides, the study’s findings limited to the specific time period, long-term monitoring and analysis would provide a more comprehensive understanding of the effects of environmental factors on the photosynthetic vitality of large old *Z. serrata*. Moreover, the photosynthetic vitality may be the result of various tangible and intangible management efforts reflected over a long period with respect to the conservation specificity of large old trees. As the scope and level of management vary depending on the target site, it is necessary to analyze the timing, method, and degree of management at a precise scale. Furthermore owing to their nature, large old trees have biomechanically (i.e. allometrically) grown by adapting to the climate of the region for a long time, and physiological reactions can develop in various ways depending on the structural and anatomical characteristics of aging. In the future, considering these points comprehensively, it is necessary to accurately analyze the causal relationship of the physiological response of large old trees and establish fundamental conservation measures accordingly.

## Materials and methods

### Study sites and large old *Z*. *serrata*

Research data were collected from large old *Z. serrata* found in Daejeon Metropolitan City (N36° 21′ 1.17″, E127° 23′ 4.43″), Gongju-si (N36° 21′ 37.06″, E127° 8′ 30.12″), and Geumsan-gun (N36° 11′ 41.48″, E127° 28′ 33.72″), which are basin-shaped urban areas in the central part of the Korean Peninsula located in the far eastern part of the Northern Hemisphere. Field surveys were conducted between April and September 2022 on 25 large old *Z. serrata* trees with a mean age of 274 years, mean tree height of 14.4 ± 2.6 m, and mean diameter at breast height (DBH) of 143.8 ± 37.6 (Table 4).

**Table 4:** Summary of the size information and the statistics for field-measured large old *Z. serrata* trees.

Tree no.	Estimated age (year)	DBH (cm)	Height (m)	Crown width (m)
1	331	132.5	15.4	S.N. 23.3/E.W. 20.8
2	151	106.0	11.0	S.N.12.3/E.W. 13.5
3	131	118.5	16.7	S.N.22.0/E.W.23.5
4	319	184.0	13.7	S.N. 13.4/E.W. 9.6
5	229	165.5	12.4	S.N. 14.0/E.W. 10.0
6	231	175.0	12.9	S.N. 19.4/E.W. 25.1
7	231	169.5	13.6	S.N. 26.0/E.W. 16.6
8	416	161.0	19.6	S.N. 20.8/E.W. 23.3
9*	394	130.0	18.1	S.N.20.0/E.W.19.8
10*	376	124.0	16.6	S.N.16.2/E.W.20.4
11	359	104.0	16.2	S.N. 12.6/E.W. 15.5
12	189	143.5	10.7	S.N. 24.3/E.W. 24.2
13	151	141.5	17.7	S.N. 24.6/E.W. 23.0
14	89	109.5	15.7	S.N. 22.5/E.W. 21.3
15	89	124.0	16.2	S.N. 15.0/E.W. 18.0
16	239	151.7	14.4	S.N. 11.9/E.W. 11.3
17	220	102.0	11.9	S.N. 12.4/E.W. 11.3
18*	300	98.0	13.2	S.N. 14.2/E.W. 12.0
19	200	112.0	10.2	S.N. 11.0/E.W. 10.0
20	250	181.0	14.1	S.N. 13.0/E.W. 15.0
21	188	91.5	12.1	S.N. 13.7/E.W. 13.1
22	468	177.5	12.4	S.N. 15.4/E.W. 15.6
23	539	252.5	16.1	S.N. 22.4/E.W. 22.2
24	539	187.5	10.9	S.N. 14.5/E.W. 15.5
25	219	153.5	17.0	S.N. 20.1/E.W. 22.5
Mean ± SD	274 ± 127.3	143.8 ± 37.6	14.4 ± 2.6	S.N. 17.4 ± 4.8/E.W.17.3 ± 5.1
Min.-Max.	89.0–539.0	91.5–252.5	10.2–19.2	S.N. 11.0–26.0/E.W. 9.6–25.1

SD, min, and max stand for the standard deviation, minimum, and maximum, respectively, *DBH* diameter at breast height, *S.N* South-North, *E.W*. East–West, Estimated age = The mean age of large old *Z. serrata* was indirectly estimated based on the age information described on the on-site board of protected *Z. serrata.*

*Estimated by applying the DBH estimation formula and the coefficient of *Z. serrata* calculated by Son et al.^49^.

**Figure 5:**
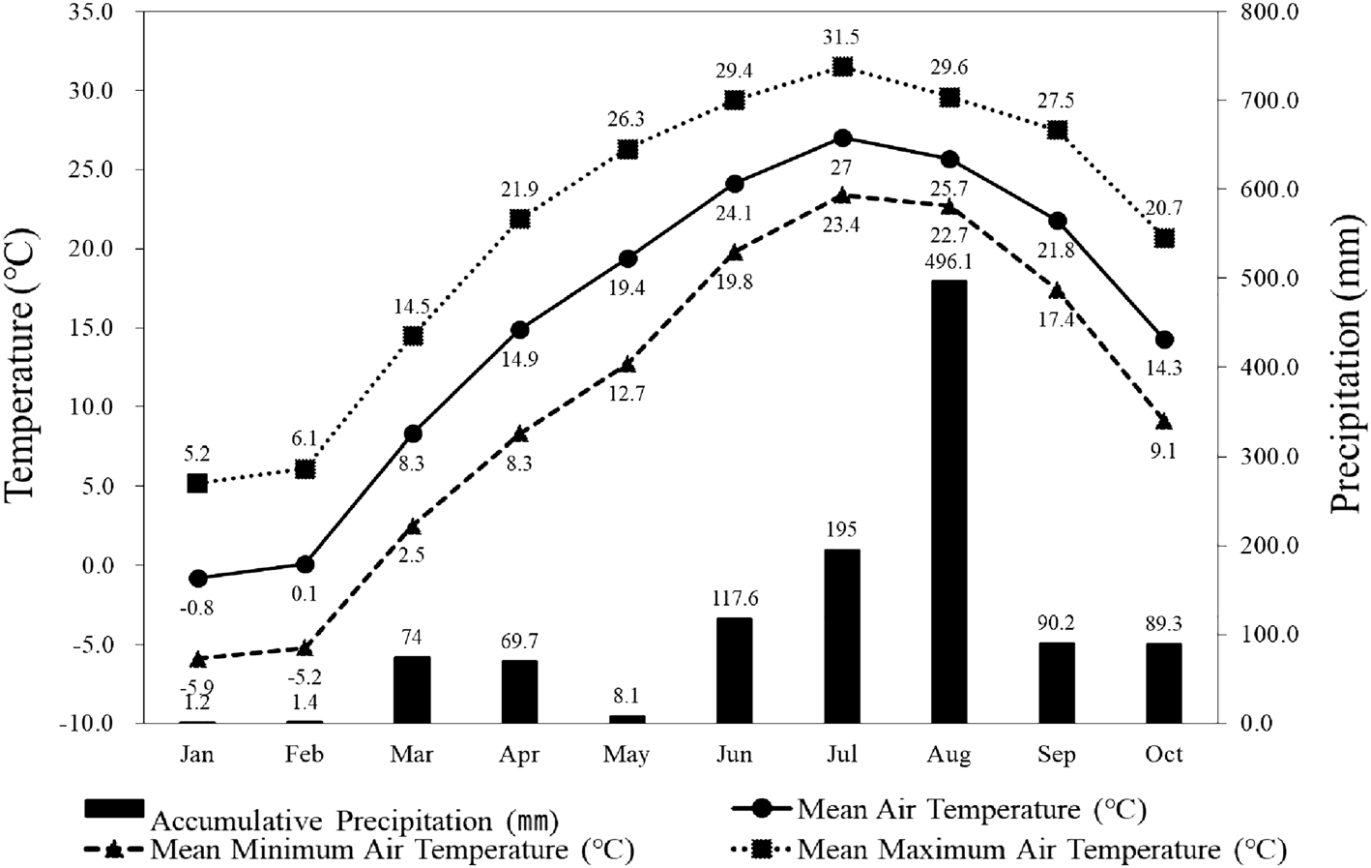
Monthly air temperature (maximum, minimum, and mean) and accumulative precipitation at the study sites of large old *Z. serrata*.

The study sites have a typical temperate continental climate with four distinct seasons and are hot during summer and cold during winter. In the past 30 years (1991–2020), the mean annual air temperature was 13.1 °C; the mean monthly air temperature during the hottest month (August) was 26.0 °C and that in the coldest month (January) was – 1.0 °C, with an annual range of 27.0 °C (Fig. 5). The mean annual precipitation was 1351.2 mm, with 60% falling between June and August and 5–10% during winter. Between January and October 2022, the mean air temperature in the study sites was 14.5 ℃, with January having the lowest air temperature of – 1.0 ℃, while the mean air temperature in July was 26.3 ℃ and the mean maximum air temperature was the highest at 30.8 ℃. The mean air temperature during spring (March–May) was 14.2 ℃, which was 1.0 ℃ higher than the national mean of 13.2 ℃ and 2.3 ℃ higher than the national mean for an average year, indicating that the year 2022 had the hottest spring temperature in the past 50 years^43^.

### Measurements of photosynthetic characteristics

A portable photosynthesis measurement system (GFS-3000, Heinz Walz GmbH, Effeltrich, Germany) was used to measure photosynthetic characteristics (gas exchange) between 8:30 AM and 11:30 AM once a month on a clear, windless day. The measurement period was from May (spring) to September (fall) 2022, which is the growth season of *Z. serrata*. For the measurements, three branches were collected from the southern part of the lower canopy, and one fully expanded leaf in full sunlight (the second or third leaf from the top) from each branch was measured. Each leaf was measured four times at 10 s intervals, and the results were recorded. The branches were collected using high-branch scissors, and the measurements were completed within 40 min of collection^44, 45^. Data collection and sampling of plants materials from large old trees in this study was conducted legally in accordance with Article 13–3 of the Forest Protection Act of the Republic of Korea, and permissions for collection for large old trees managed by local government were obtained through the exchange of official documents.

The photosynthesis measurement system was used to measure the photosynthetic physiological response parameters, such as maximum assimilation rate (A_max_, μmol·m^–2^·s^–1^), transpiration rate (E, mmol·m^–2^·s^–1^), stomatal conductance (g_s_, mmol·m^–2^·s^–1^), and water use efficiency (WUE, μmolCO_2_·mmolH_2_O^–1^). During the measurements, the temperature and RH inside the chamber was set to 25 ± 0.5 ℃ and 60 ± 1%, respectively. Moreover, CO_2_ supplied from the CO_2_ cylinder was used with a CO_2_ concentration set to 400 μmol·mol^–1^^46^ and a flow rate set to 600 μmol·s^–1^. In this study, photosynthesis was measured using an LED light source attached to the photosynthesis measurement system. However, considering that plant water status, water use pattern, and gas exchange in leaves are closely associated with climatic factors and soil moisture status and that they show diurnal and seasonal fluctuations^28, 47^, the measurements were taken after fixing the photosynthetic photon flux density (PPFD) to 1000 μmol·m^–2^·s^–1^. A_max_ was measured after 20 min of acclimation to saturation light. WUE is calculated as a ratio of A_max_ to E (A_max_/E). To analyze the photosynthetic characteristics according to climatic environmental factors, photosynthetic active radiation (PAR, μmol·m^–2^·s^–1^), leaf temperature (T_leaf_, ℃), air-to-leaf vapor pressure deficit (VPD, kPa), intercellular CO_2_ concentration (C_i_, ppm), and ambient CO_2_ mole fraction (ppm) were also measured using equipment built into the photosynthesis measurement system and sensor attached to the outside of the system.

### Measurement of physical environmental factors

To assess physical environmental factors, a field survey form was used to collect separation distance from the road, depth of soil covering, impervious surface ratio, growth space ratio, surface coverage type of growth space, and tree information. The information was recorded focusing on factors associated with growth space that may have a potential impact on tree growth (Table 5).

**Table 5:** Description of basic information of large old *Z. serrata* and growth space data collected by field survey.

Data item		Description of variables
Tree information	DBH^a^	Mean 143.8 ± 37.6 cm
	Tree height	Mean 14.4 ± 2.6 m
	Tree crown width	South–North: mean 17.4 ± 4.8 m, East–West: mean 17.3 ± 5.1 m
Growth space	Mean separation Distance from the road^b^	< 0.5 m (4.2%), 0.5–1 m (25.0%), 1–2 m (12.5%), 2–3 m (16.7%), ≥ 3 m (41.7%)
	Growth space ratio^c^	0–100% (16.0%), 100–200% (76.0%), 200% (8.0%)
	Surface coverage type	Bare land (37.8%), concrete (31.1%), gravel (13.3%), others (8.9%), woodchip (6.7%), grass (2.2%)
	Impervious surface ratio^d^	0–20 (16.0%), 21–49 (16.0%), 50–79 (36.0%), ≥ 80 (32.0%)
	Depth of soil covering	≤ 20 (16.0%), 50–60 (68.0%), ≥ 100 (16.0%)
	Presence of reinforcing stone walls	Present (40.0%), absent (60.0%)

^a^Diameter at breast height (1.2 m above ground level) were measured.

^b^Mean separation distance from the tree to the nearest road.

^c^Ratio of growth space area relative to the crown width area (if the growth space is smaller than the crown width area, “ − 1” was multiplied by the decreased ratio to convert it to a negative number).

^d^Ratio of impervious surface within the crown width area.

### Data analysis

Descriptive statistical analysis was performed on the basic specifications of large old *Z. serrata* and the physical environmental factors; correlations with photosynthetic characteristics were analyzed to derive Pearson’s correlation coefficients (r), standardized covariance, to calculate the effect size of independent variables on the dependent variable. A simple regression analysis based on a general linear model was performed on variables confirmed to be significant. After assessing the goodness of fit of the model using least squares approximation, the F ratio, the ratio between the model and observed values, was derived to assess the explanatory power of the regression model.

Among the independent variables, the physical environmental factors were divided into two categories depending on their presence or absence in the environment. The first category included adjacent land type, such as agricultural, residential, roads, barriers, and reinforcing stone walls and were analyzed and compared using a non-parametric Wilcoxon rank-sum test. The second category included surface type, which was divided into three categories of bare land, mulching material (including gravel, woodchip, and others), and concrete, and the mean values among the three groups of surface types were compared and analyzed using a non-parametric Kruskal–Wallis H test. If differences among the three groups were found, the post-hoc test was performed to determine the source of such differences. Each variable for the physical growth environment was analyzed by descriptive statistical analysis. All statistical analyses were performed using R 4.2.2^48^.

### Supplementary Information

Supplementary Table S1.

## Data Availability

The datasets generated during and/or analyzed during the current study are available from the corresponding author upon reasonable request.
