# Supplementary material for: Photosynthetic responses of large old Zelkova serrata (Thunb.) Makino trees to different growth environments
Source: Sci Rep. 2023 Nov 18;13:20205. doi: 10.1038/s41598-023-47561-3 (PMC10657411; doi:10.1038/s41598-023-47561-3)
Supplement: Supplementary file 1 — Supplementary Table S1. [file 41598_2023_47561_MOESM1_ESM.docx]

**Supplementary Information**

**Photosynthetic Responses of Large Old *Zelkova serrata* (Thunb.) Makino Trees to Different Growth Environments**

Ji Sun Jung^1^, Gwang Gyu Lee^1^, Ji Won Son^1*^, Chae Won Kim^2^, Yoo Jin Ahn^1^

^1^ Natural Heritage Center, National Research Institute of Cultural Heritage, Daejeon, Republic of Korea

^2^ Korea National University of Cultural Heritage, Buyeo, Republic of Korea

Correspondence and requests for materials should be addressed to J.W.S.

^*^wine814@korea.kr

**Supplementary Tables**

**Table S1.** The statistics of the tree height and maximum assimilation rate (A_max_) of large old *Z. serrata* trees during the entire growth season.

| Tree no. | Tree height (m) | A_max_ (μmol·m^-2^·s^-1^) | |
| --- | --- | --- | --- |
|  |  | Mean | SD |
| 1 | 15.4 | 3.53 | 5.26 |
| 2 | 11.0 | 2.60 | 2.09 |
| 3 | 16.7 | 2.32 | 2.30 |
| 4 | 13.7 | 5.96 | 2.77 |
| 5 | 12.4 | 2.97 | 2.25 |
| 6 | 12.9 | 1.52 | 0.41 |
| 7 | 13.6 | 1.80 | 0.78 |
| 8 | 19.6 | 0.93 | 0.67 |
| 9 | 18.1 | 1.11 | 1.17 |
| 10 | 16.6 | 1.36 | 0.16 |
| 11 | 16.2 | 1.75 | 1.98 |
| 12 | 10.7 | 5.56 | 5.76 |
| 13 | 17.7 | 3.72 | 2.93 |
| 14 | 15.7 | 1.38 | 0.55 |
| 15 | 16.2 | 1.31 | 0.67 |
| 16 | 14.4 | 1.69 | 1.84 |
| 17 | 11.9 | 1.66 | 0.75 |
| 18 | 13.2 | 3.75 | 1.93 |
| 19 | 10.2 | 4.87 | 3.90 |
| 20 | 14.1 | 2.35 | 0.85 |
| 21 | 12.1 | 7.10 | 1.86 |
| 22 | 12.4 | 2.89 | 1.79 |
| 23 | 16.1 | 3.20 | 1.50 |
| 24 | 10.9 | 1.73 | 0.97 |
| 25 | 17.0 | 1.30 | 1.53 |

SD stands for the standard deviation.
